# Editorial: Rising stars in bacteria and host: 2022

**DOI:** 10.3389/fcimb.2023.1240952

**Published:** 2023-06-30

**Authors:** Naile Dame-Teixeira, Thuy Do

**Affiliations:** ^1^ Division of Oral Biology, School of Dentistry, University of Leeds, Leeds, United Kingdom; ^2^ Department of Dentistry, University of Brasilia, Brasilia, Brazil

**Keywords:** human microbiome associated diseases, microbiome & dysbiosis, host microbiome interaction, dysbioses, microbial community diversity

Identifying and supporting future research leaders in Bacteria and Host is essential for ensuring tomorrow’s innovations are driven by cutting-edge research. This Research Topic features the work of some early-career researchers who are making significant contributions to the field. The goal is to highlight their work and showcase their potential to address pressing challenges related to this important Research Topic.

The human microbiome is rich and diverse, comprising large bacterial populations, it also includes low abundant non-bacterial organisms (de Cena et al., 2021). Studying how bacteria and hosts interact is essential for understanding important aspects of human health, disease, and the environment. Human-associated microorganisms play a vital role in human health, helping to digest food, fight off infection, and regulate the immune system (Zaura et al., 2009; Consortium, H. M. P., 2012; Zaura et al., 2014; Ranjan et al., 2018). However, when there is a disruption in the balance of the human microbiota – namely dysbiosis – it interferes with the harmonious relationship and paves the way for the emergence of various diseases in the host.

Research on bacteria-host interactions is a complex and challenging field. It often requires expertise from a variety of disciplines, including clinical medicine, microbiology, and bioinformatics. Bridging the communication gap through interdisciplinary collaborations has the potential to unlock novel insights into disease mechanisms. This Research Topic showcases work that incorporated molecular techniques and animal models in bacteria-host research, which significantly broadens our understanding on how to maintain homeostasis.

A well-documented breakdown of the bacteria-host partnership is periodontitis (Marcenes et al., 2013). This is an inflammatory disease caused by a dysbiotic subgingival microbiome, frequently leading to tooth loss and all the consequences of it, such as worsened nutrition and metabolic levels, and poor quality of life. Fan et al. focused on understanding the host response to *Porphyromonas gingivalis*, a periodontitis-associated bacteria. They highlighted the significance of sialidase in the virulence of *P. gingivalis*. They found that deficiency in sialidase made it easier for macrophages to recognize the bacterium. Additionally, they discuss that the lack of sialidase results in reduced fimbria and gingipain activity, as well as a decrease in the protective capsule layer that often helps *P. gingivalis* evade immune clearance. These findings shed light on the importance of characterizing bacterial pathogenicity and its mechanisms for evading the host immune response.

The study by Peng et al. investigated the changes in the microbiota in different intestinal segments of mice with sepsis. The authors found that there was a significant decrease in the abundance of beneficial bacteria, such as Lactobacillus and Bifidobacterium, in the cecum and colon of mice with sepsis. They also found an increase in the abundance of potentially harmful bacteria, such as Bacteroides and Proteobacteria. These changes in the microbiota were associated with increased intestinal permeability and inflammation. These findings may help explain a heightened risk of sepsis-related complications, such as multi-organ failure, in patients with sepsis. Therefore targeting the microbiota may be a potential therapeutic strategy for preventing or treating sepsis.

When homeostasis of the microbial community and host is broken down, it may be reestablished through microbiome modulation strategies. As an example, studies on probiotic and immunomodulation effects of several strains have garnered considerable attention in recent years. Probiotics are live microorganisms that can confer health benefits to the host. These beneficial bacteria can play a crucial role in maintaining a balanced microbiota and promoting health. Numerous studies have demonstrated the potential of probiotics in managing various gastrointestinal conditions, such as diarrhea, irritable bowel syndrome, and inflammatory bowel disease (Seminario-Amez et al., 2017). Additionally, probiotics have shown promising results in supporting immune function, reducing the risk of respiratory infections, and even improving mental health outcomes (Dolan et al., 2016; Shahbazi et al., 2020). As the scientific understanding of the human microbiome and its interaction with the host continues to expand, the integration of probiotics into clinical practice holds significant promise for optimizing patient outcomes and improving overall health. Huang et al. combined *in vitro* and *in vivo* experiments, as well as genomic analysis to demonstrate that *Limosilactobacillus reuteri* RGW1 isolated from calf feces has the capacity to biosynthesize L-lysine, folate, cobalamin, reuterin and hydrogen peroxide. The results of this study suggest that *L. reuteri* RGW1 could be a potential probiotic for use in animals. It was shown capable of modulating the immune system by increasing the production of Th1 and Th2 cytokines, and reducing pro-inflammatory cytokines, therefore reducing intestinal inflammation. Further studies are needed to confirm these findings and to investigate its potential benefits of in humans.

Another study (First et al.) investigated the possible host immunomodulatory mechanisms that can be exploited to promote clearance of pathogens such as *Bordetella* app. from the lower respiratory tract. *Bordetella* spp. are respiratory pathogens that can cause whooping cough, parapertussis, and bronchiolitis. These pathogens are able to colonize and persist in the lower respiratory tract by manipulating the host’s VIP/VPAC2 signaling pathway. The type 3 secretion system (T3SS) is a virulence factor that *Bordetella* spp. use to deliver effector proteins into host cells. These effector proteins interfere with the function of VIP/VPAC2 receptors, which leads to increased mucus production, inflammation, and impaired clearance of bacteria from the lungs.

This study provides new insights into bacteria-host crosstalk that could provide a target for the future treatment for whooping cough as well as other infectious diseases caused primarily by persistent mucosal infections.

In this Research Topic, papers investigated the host-microbiome interactions and integrated several methods to study the context of health and disease. We believe this Research Topic will be a valuable resource to the scientific community and to those who are interested in the future of Bacteria and Host. The Research Topic showcases 4 papers that describe survival mechanisms of bacteria and their impact on the host and environment. The authors of these papers use cutting-edge approaches to gain new insights into the complex interactions between bacteria and their hosts. This research has the potential to improve our understanding of bacterial diseases and lead to the development of new and effective treatments. We hope that it will inspire others to pursue research in this field and help to accelerate the pace of discovery.

## Author contributions

ND-T and TD contributed to the conception of the work; drafted the manuscript and revised it critically; approved the content and agreed to be accountable for all aspects of the work.
